# Anti-cancer therapeutic strategies based on HGF/MET, EpCAM, and tumor-stromal cross talk

**DOI:** 10.1186/s12935-022-02658-z

**Published:** 2022-08-19

**Authors:** Khadijeh Barzaman, Rana Vafaei, Mitra Samadi, Mohammad Hossein Kazemi, Aysooda Hosseinzadeh, Parnaz Merikhian, Shima Moradi-Kalbolandi, Mohammad Reza Eisavand, Houra Dinvari, Leila Farahmand

**Affiliations:** 1 Recombinant Proteins Department, Breast Cancer Research Center, Motamed Cancer Institute, ACECR, Tehran, Iran; 2 Department of Immunology, School of Medicine, Iran University of Medical Sciences, Tehran, Iran; 3 Department of Clinical Pathology, Faculty of Veterinary Medicine, University of Tehran, Tehran, Iran; 4 ATMP Department, Breast Cancer Research Center, Motamed Cancer Institute, ACECR, Tehran, Iran

**Keywords:** EpCAM, HGF/MET, NF-κB, Wnt, MAP/PI3K/mTOR

## Abstract

As an intelligent disease, tumors apply several pathways to evade the immune system. It can use alternative routes to bypass intracellular signaling pathways, such as nuclear factor-κB (NF-κB), Wnt, and mitogen-activated protein (MAP)/phosphoinositide 3-kinase (PI3K)/mammalian target of rapamycin (mTOR). Therefore, these mechanisms lead to therapeutic resistance in cancer. Also, these pathways play important roles in the proliferation, survival, migration, and invasion of cells. In most cancers, these signaling pathways are overactivated, caused by mutation, overexpression, etc. Since numerous molecules share these signaling pathways, the identification of key molecules is crucial to achieve favorable consequences in cancer therapy. One of the key molecules is the mesenchymal-epithelial transition factor (MET; c-Met) and its ligand hepatocyte growth factor (HGF). Another molecule is the epithelial cell adhesion molecule (EpCAM), which its binding is hemophilic. Although both of them are involved in many physiologic processes (especially in embryonic stages), in some cancers, they are overexpressed on epithelial cells. Since they share intracellular pathways, targeting them simultaneously may inhibit substitute pathways that tumor uses to evade the immune system and resistant to therapeutic agents.

## Introduction

The mesenchymal–epithelial transition factor (MET) gene is expressed on the membrane that is bound to receptor tyrosine kinase (RTK). Besides, epithelial cells express essentially the MET receptor [1]. The hepatocyte growth factor (HGF), as a serum ligand, activates MET/RTK. It has been known as a mitotic factor for hepatocytes. Followed by binding it to c-Met from tumor cells, a signaling pathway is formed, which leads to proliferation, metastasis, and angiogenesis, for example in brain, gastric, and head and neck cancers [2, 3]. Also, stromal cells and fibroblasts are the main source of HGF production. Thus, HGF/MET activation can lead to numerous intracellular events, such as proliferation, survival, and inflammation pathways. Therefore, various molecules such as extracellular signal-regulated kinase 1 and 2 (ERK1/2)/mitogen-activated protein kinases (MAPKs), phosphoinositide 3-kinase (PI3K)/protein kinase B (Akt), signal transducer and activator of transcription (STAT), and nuclear factor-κB (NF-κB) are involved in it. Of note, over-activation of the HGF/MET pathway through germline MET and sporadic MET mutations or even protein over-expression increases tumorigenesis and tumor progressions in numerous cancer forms, such as renal cell carcinoma, metaplasia-dysplasia-adenocarcinoma evolution in esophageal cancer, osteosarcomas, and melanomas, as well as brain, gastric, gliomas, breast, and head and neck cancers [1, 4–6]. Moreover, the literature has shown that changes in MET are related to anti-cancer resistance in some cancers, for example, non-small cell lung cancer (NSCLC) and colorectal cancer (CRC); also, it is associated with worse prognosis and aggressiveness [7, 8]. On the other hand, various epithelial tissues expressed the epithelial cell adhesion molecule (EpCAM). It is a 40-kD transmembrane glycoprotein that consists of 341 amino acids. Its structure includes the extracellular domain (EpEX), single transmembrane domain, and intracellular domain (EpICD) [9]. Also, EpCAM is a cell surface marker on many kinds of stem cells and progenitor cells [10, 11]. Previous studies have demonstrated that EpCAM is involved in cell junction via interacting with several important cell adhesion molecule (CAM) junctions [9]. In the 1970s, after administration of cancer cells to the mic, EpCAM was identified as a novel tumor-specific cell surface antigen. Also, it is highly expressed in many kinds of epithelial carcinomas. Thus, it correlates with tumorigenesis, metastasis, and cancer stem cells [12]. Also, some antibodies considering that can target EpCAM are developed [13]. Since EpCAM and HGF/c-Met are involved in important signaling pathways in various cancers, the purpose of this study is to elucidate the interplay between these molecules, tumor microenvironment, and intracellular pathways. The reason is that simultaneously targeting these two molecules may boost the efficacy of cancer therapies.

## Interplay HGF/c-Met and tumor stroma

Tumor stroma consists of extracellular matrix (ECM) and various cells, such as fibroblasts, inflammatory and endothelial cells. This composition significantly impacts tumor initiation and progression [14]. Cross talking between tumor and stromal cells results in a suitable microenvironment for tumor growth and metastasis [14, 15]. Fibroblasts are the most frequent cells in the tumor stroma. They have important roles in the maintenance of ECM and adjacent epithelial homeostasis via direct stromal-epithelial contact and the secretion of cytokines [15]. Normal fibroblasts turn into cancer-associated fibroblasts (CAFs), followed by the creation of neoplastic transformation of epithelia. Then, they boost their capacity to promote the malignant process through the production of growth factors and inflammation factors [16, 17]. HGF and MET are expressed by stromal and malignant cells, respectively. Thus, when HGF is coupled with its proto-oncogene receptor, c-Met leads to epithelial phenotype transformation and the acquisition of a migratory phenotype of noncancerous cells. It is a critical, widely documented phenomenon in the transformation of neoplastic features regarding the progression of various cancers [18]. Therefore, HGF creates a microenvironment through interaction between cancerous cells and adjacent stroma, increasing the further development and invasiveness of cancer [19]. HGF facilities cell detachment from the primary tumor. Then, they are infiltrated via the surrounding stroma favoring the pathways, leading to degradation of ECM [18].

### Cross talk between HGF/c-Met and immune responses

**Fig. 1:**
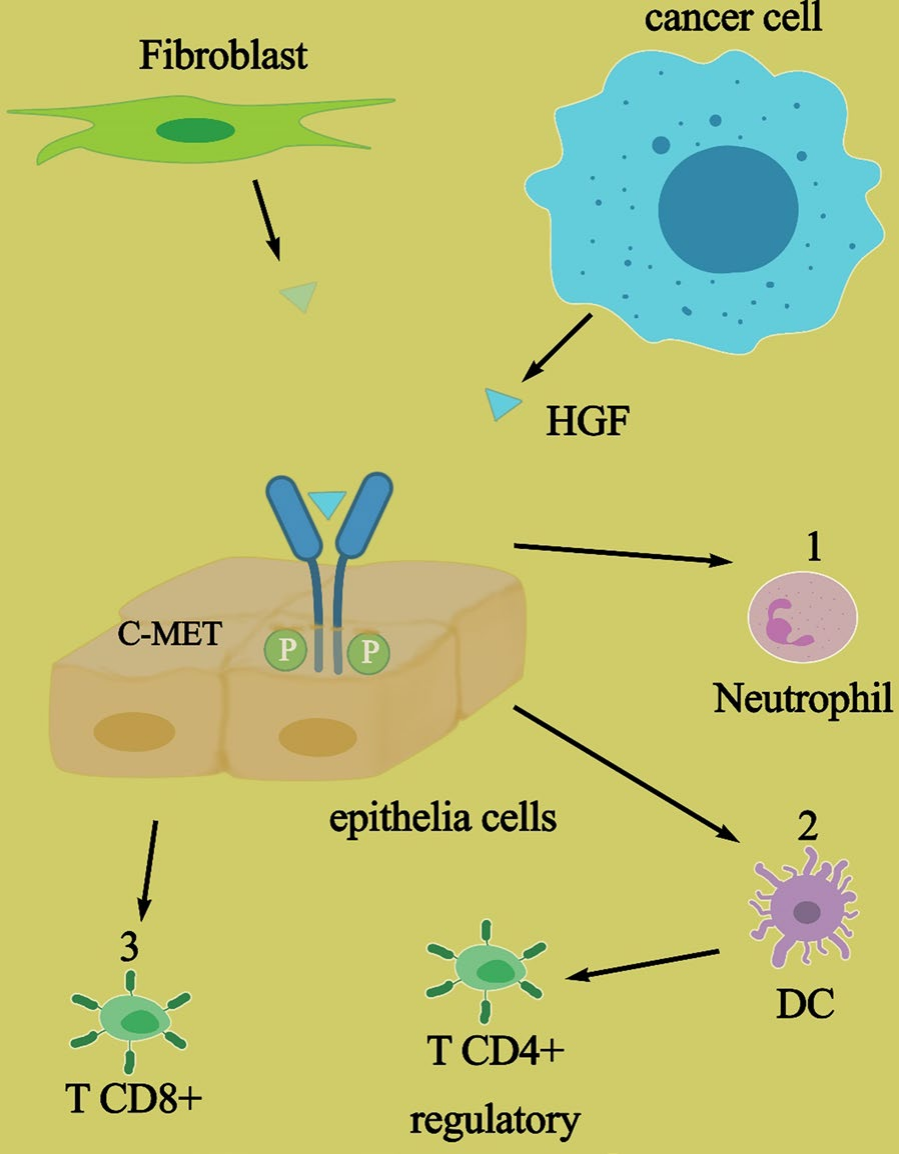
Controversial roles of HGF/c-Met in the immune system: (1) chemoattraction of neutrophils to tumor site; (2) activated Dc presenting TAA, boost TCD4+ regulatory cells, and anti-tumoral immune response decreased; and (3) TCD8+ activation, which led to anti-tumoral immune response

Previous results have demonstrated that HGF/MET axis impacts immune responses [1, 20]. Although its effects are unclear, the migration of T and B lymphocytes is controlled by HGF. Also, it can counteract the anti-inflammatory effect of transforming growth factor (TGF) [21–24]. In an experimental animal model of auto-inflammatory disease [experimental autoimmune myocarditis (EAM)], for instance, a greater amount of HGF is conversely associated with inflammation and fibrosis [1, 23]. Furthermore, HGF, in cooperation with other hematopoietic stimuli, is able to increase all types of precursors [25]. Its receptor, MET, is known as a tumor-associated antigen (TAA). Thus, MET can be recognized by CD8 cytotoxic T cells. This mechanism can initiate immune system activation against cancer cells overexpressing MET [26]. MET has significant effects on the immune system via dendritic cells (DCs). DCs, which present TAA to T cells, can induce the activation of regulatory T cells (CD4+), controlling cytotoxic CD8+ T cells. Thus, the HGF/MET axis can boost this mechanism. It showed that this pathway could be targeted for cancer immunotherapy [1]. DCs affected by HGF can induce an increase in T regulatory, interleukin 10 (IL-10), and transforming growth factor β (TGF-β). Also, they increase IL-17-producing lymphocytes [27, 28]. Therefore, this process leads to the inhibition of the immune response [1]. Furthermore, HGF/MET can affect the immune system via granulocytes. The literature has shown that MET deletion in neutrophils leads to the enhancement of tumor growth and metastasis. Thus, MET can play an essential role in chemoattraction and neutrophil-mediated cytotoxicity. The tumor-derived tumor necrosis factor (TNF) or other inflammatory factors can induce MET in human neutrophils, leading to transmigration neutrophils across an activated endothelium, and free radical production results in cancer cell killing. Therefore, it should be taken into consideration that treating cancer patients with MET inhibitors can lead to defective chemotaxis of neutrophils, and tumor cells can escape from tumor killing [29]. Indeed, in some cases, the HGF/MET axis is crucial for cancer cell survival, and, in other cases, it has anti-cancer effects [1]. Thus, it is complicated to target just the HGF/MET pathway in cancer therapy (Fig. 1).

#### Interplay HGF/c-Met with cytokines

Tumor cells and CAfs, in tumor stromal, produce several cytokines such as IL-6, IL-8, IL-10, monocyte chemoattractant protein 1 (MCP-1), and regulated on activation, normal T cell expressed and secreted (RANTES) [30]. IL-6 is one of the important cytokines, which is well known as a pro-inflammatory cytokine. It plays key roles in some processes, such as B and T differentiation, induction of acute-phase mediators, hematopoiesis, tumor cell proliferation, and increased angiogenesis [31, 32]. There are direct and indirect correlations between disease progression and response to therapeutic agents, respectively, with IL-6 levels [33]. The IL-6/JAK2/STAT3 pathway is activated by coupling IL-6 with its cell-surface receptor (IL-6R) and a common cytokine-receptor signal-transducing subunit (gp130) [34–36]. Also, in various solid tumors, HGF and IL-6 play key roles in the phenotype modulation of cancer cells [14, 37]. Ding et al. demonstrated that HGF cooperated with IL-6; also, they showed that the increased level of MET led to the differentiation of normal fibroblast to CAFs in gastric cancer (GC) [14]. To et al. showed that the interaction between HGF and IL-6 was conducted via two ways involved in the invasion of a lung cancer cell line in vitro [38]. They demonstrated that IL-6 was able to stimulate A549 lung adenocarcinoma and increase messenger RNA (mRNA) expression of c-Met/HGF. Also, their results demonstrated that the production of matrix metallopeptidase 2 (MMP-2) and MMP-9 was increased when there was co-stimulation with HGF and IL-6. Thus, it led to an extra effect on tissue invasion [38]. On the other hand, another pro-inflammatory cytokine is IL-8, which has numerous activities such as the migration of neutrophils, monocytes, tumor cell proliferation, and metastasis [32, 39]. Additionally, it is known as an important cytokine involved in the angiogenesis process [40]. Furthermore, RANTES and MCP-1 are other chemokines that have important roles in the migration of normal and malignant cells [33, 41, 42]. Also, IL-10 is one of the cytokines involved in the immunosuppressive process. Thus, it is considered a protecting cancer cell agent; also, it is frequently produced by tumor cells [33]. Previous studies have shown that in bone marrow stromal cells (BMSCs), production of HGF results in the production of IL-11, IL-10, IL-6, IL-8, stromal cell-derived factor (SDF)-1α, and vascular endothelial growth factor (VEGF) [43]. Another role of HGF/c-Met is to decrease the expression of interferon γ (IFN-γ), TGF-β, and TNF-α in a dose-dependent manner [44]. Boissinot et al. demonstrated that in the serum and bone marrow plasma of polycythemia vera (PV) patients, the levels of HGF, IL-11, and tissue inhibitor of metalloproteinase 1 (TIMP-1) increased. Also, they showed that paracrine and autocrine feedback loops were the main ways in which BMSCs and glycophorin A+ (GPA) erythroblasts are involved, and HGF and IL-11 directly affected the production of each other [45].

In conclusion, these network complex connections of signals and mediators are in ECM and have great impacts on cell proliferation and function deviation. Focusing on the management of immunosurveillance, angiogenesis, and key factors produced by tumor cells and CAFs (as prominent cells in the tumor microenvironment) can open ways to increase the efficacy of tumor therapy.

## Interplay EpCAM with cancers

EpCAM (or CD326A) is a well-known pan-epithelial differentiation antigen expressed on a vast number of epithelial tissues. It is also involved in cell signaling, proliferation, differentiation, and migration. EpCAM is overexpressed on the basolateral in the epithelial malignancies’ surface of all human carcinomas of various origins [46, 47]. For example, in hepatic malignancies, overexpression of EpCAM relates to poor prognosis because it activates proto-oncogene myelocytomatosis (c-Myc). Thus, it leads to tumor progression [48]. Also, CD326 is considered as a promising target for anti-cancer therapy because of being a part of the molecular network of oncogenic receptors. Also, EpCAM plays an important role in the suppression of anti-tumor immunity. Thus, immune-based therapies are applied to target EpCAM [46].

### Cross talk between EpCAM/EpCAM and immune responses

Since EpCAM is a common TAA and can affect T-cell immune responses, Ziegler et al. demonstrated that it could be considered an immune target in colon cancer [49]. EpCAM also leads to IL-4 dominated T helper 2 (Th2) responses. Therefore, Th1-inducing conditions are rarely dominant. They also showed that intra-tumoral expression of cytokines of the IL-12 family and IFN-γ (which are caused by induction Th1 and lead to inhibition of tumor growth) diminished. In return, EpCAM as a human TAA can cause tumor immune evasion via Th2 responses’ development [49]. For example, in ovarian cancer progression, the immune system plays pivotal roles throughout cytokine and chemokine signaling pathways in drug resistance [50, 51].

### Interplay EpCAM with cytokines

As mentioned above, the two most important cytokines, IL-6 and IL-8 (CXCL8/IL-8), are involved in various spectrum cellular pathways responsible for the proliferation, metastasis, or tumor cell survival. Also, the data showed that IL-6 and IL-8 affect the expression of EpCAM. Bonneau et al. demonstrated that IL-8 could impact epithelial-mesenchymal transition (EMT) in ovarian and breast cancer (BC) cells. Also, in patients with ovarian cancer, the level of EpCAM can be considered as a predictor of poor prognosis [46]. Additionally, according to the type and/or signaling pathway, AP-1, NF-κB, and C/EBPb transcription factors are involved in IL-8 regulation [52]. In an experiment conducted by Narendra et al. [53], it was illustrated that EpCAM has a correlation with IL-8 in primary BC. They also showed that when EpCAM was downregulated in BC cell lines, IL-8 expression decreased. Consequently, phosphorylation of NF-κB family member RELA increased, while IκBα protein expression decreased. Therefore, EpCAM induces activation of NF-κB, followed by modulation of IL-8 expression at baseline and IL-1β stimulation. Although the data showed that the EpCAM signaling has roles in the modulation of BC invasion, to clarify the molecular mechanism of EpCAM, further study should be conducted to apply appropriate molecular therapies to boost efficacy targeting of EpCAM [53].

In conclusion, IL-8 as a CXC chemokine produced by various cell types is well-known as a potent angiogenic factor through paracrine and autocrine routes in tumorigenesis. Thus, it can be considered as an intervention in cancer metastasis [54]. Furthermore, it plays roles in diverse normal physiological processes, including wound healing and abnormal processes such as cancer metastasis. IL-8 is produced by a large number of solid tumor types and related to inflammatory cells such as neutrophils. On the other hand, the literature has indicated that endothelial cells secret IL-6 that leads to tumor growth enhancement [55]. For example, Shi et al. demonstrated that IL-6 could contribute to the regulation of VEGF and angiogenesis in GC. They showed that IL-6 was an inducer for VEGF expression, which boosted angiogenesis in GC [56]. As mentioned above, both HGF/c-Met and EPCAM signaling pathways are involved in the production of IL-6 and IL-8. Therefore, targeting both of them simultaneously may have significant effects on cancer therapy via direct and indirect effects on angiogenesis. Consequently, cancer patients might benefit from approaches targeting HGF/c-Met and EpCAM (Table 1).

**Table 1:** List of cytokines

Cytokine	Chief cell source	Chief cell target	Biological effect
IFN-γ	T helper1, T CD8+, NK cells	MQ, B, T cells	Increased antimicrobial function of MQ, isotype switching of B cells to IgG, Th1 differentiation
TNF-α	MQ, NK and T cells	Endothelial, neutrophil and…	Coagulation and inflammation of endothelial cells, activation of neutrophyils
TGF-β	T regulatory cells, MQ	T, B cells, MQ and fibroblast	Inhibition of T cells proliferation and effector function, inhibition of B cell proliferation, inhibition of MQ activation, increased synthesis of collagen in fibroblast cells
IL-1β	MQ, DC, fibroblast and endothelial cells	Endothelial cells, hypothalamus	Coagulation and inflammation of endothelial cells, induction of fever by hypothalamus
IL-4	T helper2, mast cells	B, T cells, MQ, and mast cells	IgE isotype switching of B cells, Th2 differentiation, inhibition of IFN-γ-mediated antimicrobial activation of MQ, In vitro proliferation of mast cells
IL-6	MQ, endothelial, T cells	Liver, B cells	Protein synthesis of acute phase by liver, proliferation of B cells (antibody producing cells)
IL-10	MQ, T regulatory cells	MQ and DC	Inhibition of MHC II, co-stimulators and IL-12 expression
IL-11	–	–	Production of platelet
IL-12	MQ, DC	T, NK cells	Differentiation of Th1 cells, IFN-γ synthesis and increased cytotoxic activity of NK and T cells
IL-17	T cells	Endothelial, MQ, epithelial cells	Increased production of chemokine by endothelial cells, Increased production of chemokine and cytokine by MQ, production of GM-CSF and G-CSF

Reference: cellular and molecular immunology [220]

*GM-CSF* granulocyte-macrophage colony-stimulating factor, *G-CSF* granulocyte colony-stimulating factor

IL-8 is a chemokine which secreted by MQ and attracts neutrophils

Monocyte Chemoattractant Protein-1 (MCP1): is a chemokine which regulate infiltration of MQ

## NF-κB signaling pathway in cancer

**Fig. 2:**
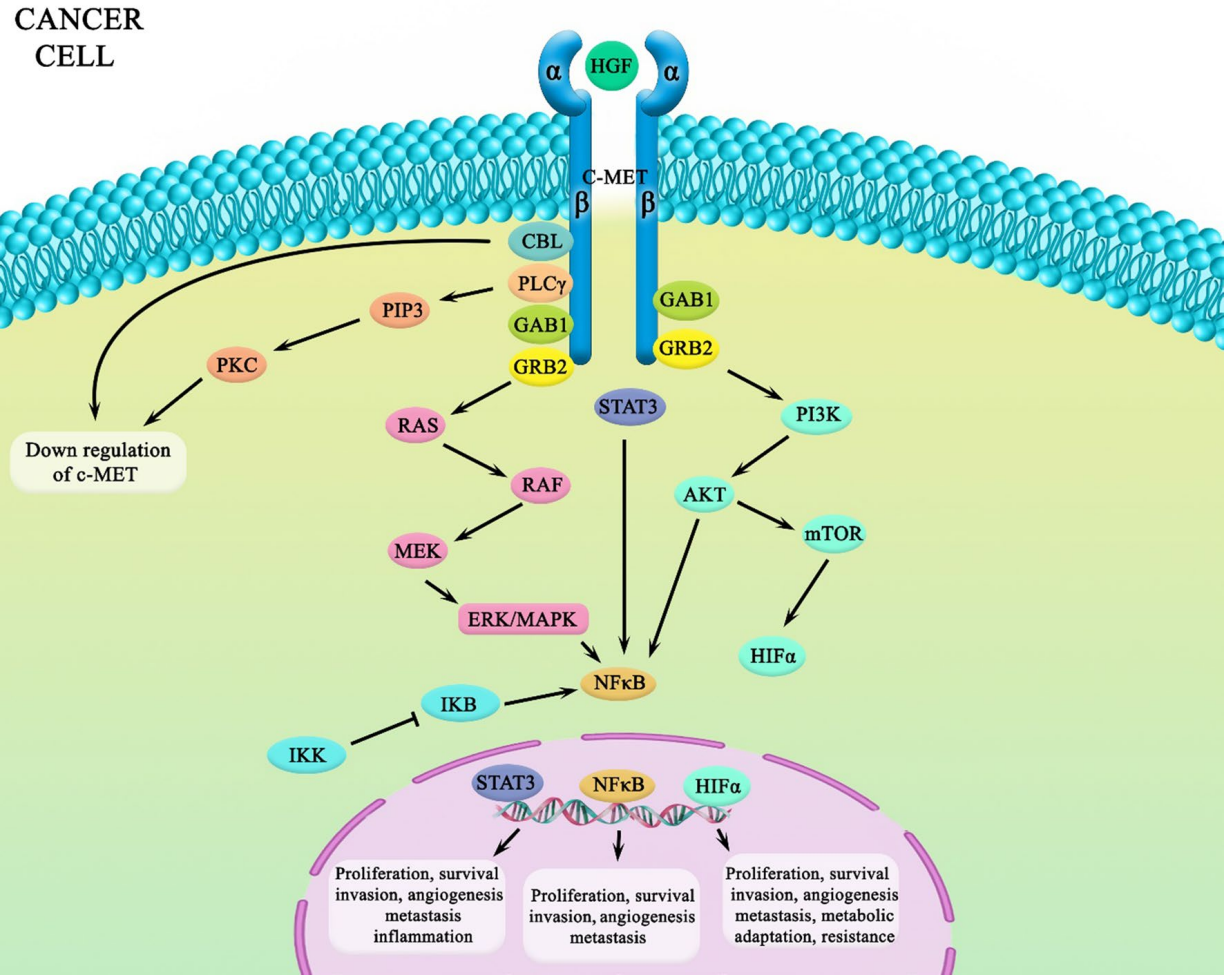
C-Met can activate NF-κB in some cancer cells. By the way, indirect interactions can be assumed from experimental data. C-Met can activate STAT3 and PI3K/Akt, which are upstream and activators of NF-κB

NF-κB is considered as a pleiotropic transcription factor. Also, numerous processes, inflammation, innate immunity, apoptosis, and cell proliferation, for instance, are mediated by it [57]. Furthermore, NF-κB is involved in various cancers, such as BC, via several stimulators, including various pro-inflammatory cytokines (IL-1β and TNF-α), growth factors (epidermal growth factor [EGF]), DNA-damaging agents (radiation), and oncogenes (rat sarcoma [RAS]) [57–59]. Also, NF-κB is a key regulator of several genes, including MMP-9, COX2, c-Myc, cyclin-D, etc. [60]. Thus, it promotes proliferation, migration, and metastasis in cancer cells [1]. IKK and IKB are positive and negative regulators of NF-κB, respectively (Fig. 2) [60]. In some cancers, such as BC, NF-κB is overexpressed [61]; NF-κB not only is involved in the proliferation and development of BC cells but also leads to resistance to some drugs used in cancer therapy such as anti-epidermal growth factor receptor (EGFR) drugs [62]. Furthermore, NF-κB plays a key role in EMT by regulating important molecules, such as MMP-9 [63]. It also protects cancer cells from apoptosis by induction of anti-apoptotic proteins, such as BCL2, BCLxL, XIAP, and so on [60, 63, 64]. NF-κB has interactions and cross talk with many intracellular molecules (e.g., NF-κB with estrogen receptor [ER] and progesterone receptor [PR] signaling pathways) [61, 65]. We are going to discuss the association between NF-κB and HGF/c-Met and EpCAM in the following sections.

## Cross talk NF-κB and HGF/c-Met

Only few studies have shown that c-Met is upstream of NF-κB in BC [66], glioma [67], and renal cancer cells [68]. It can be mentioned that c-Met may activate NF-κB by activating other molecules and pathways. For example, PI3K/Akt and ERK/MAPK are downstream pathways of c-Met [69] and can activate NF-κB [12]. STAT3 activates NF-κB in BC cells [65], and c-Met promotes proliferation and migration by triggering STAT3 in these cells [62]. Hence, it can be assumed that c-Met activates NF-κB through STAT3, PI3K/Akt, and ERK/MAPK pathways. In other cancer cells, studies suggest more cross talk between NF-κB and c-Met. It was shown that blocking c-Met/HGF (C-Met ligand) interaction would suppress MMP-9 activity in lung cancer [70]. As you can see, there is no conclusive evidence of an interaction between NF-κB and c-Met in cancer cells, especially in BC cells; hence, more studies are needed to be done to clearly identify this interaction.

## Cross talk NF-κB and EpCAM

The results showed that NF-κB-EpCAM was co-overexpressed in the nucleus of BC cells [71]. Downregulation of EpCAM was followed by downregulation of NF-κB in these cells [71]. As can be seen, the results from NF-κB-EpCAM and/or c-Met and NF-κB in BC cells are limited. The only way to find the possible interaction of NF-κB-EpCAM and c-Met-NF-κB in BC cells is to find a common molecule in the upstream or downstream of NF-κB-EpCAM and/or c-Met. Hence, the relation between NF-κB-c-Met or EpCAM may indirectly assume. For example, in BC cells, NF-κB regulates c-Myc expression [60, 72]; on the other hand, EpCAM-c-Myc is co-overexpressed in these cells [72]. Thus, it could be concluded that NF-κB-EpCAM has cross talk, which can regulate c-Myc expression. MMP-9 is another protein, which may explain the possible cross talk between NF-κB-EpCAM. MMP-9 is regulated by NF-κB [60] and promotes EpCAM activity in BC cells [73]. Hence, it can be concluded that NF-κB could regulate EpCAM through MMP-9. Expression of EpCAM is linked to COX2 expression [73], and COX2 is another target of NF-κB in BC cells [73]. The mentioned conclusions are possible here. As mentioned before, NF-κB is downstream of PI3K/Akt and ERK/MAPK pathways, and targeting NF-κB could be an option to block these pathways. PI3K/Akt and ERK/MAPK are downstream of several important receptors, such as EGFR, insulin-like growth factor (IGF-IR), and vascular endothelial growth factor receptor (VEGFR). Studies have shown that blocking NF-κB increased the sensitivity of cancer cells to therapy-induced apoptosis [63, 74]. Blocking NF-κB prevents tumor formation in mice or suppresses established tumors in these animal models [63]. Other results showed that inhibiting NF-κB signaling decreased progesterone-induced proliferation in BC cell lines [63]. Because of the lack of enough data on the interaction of NF-κB-EpCAM and c-Met-NF-κB in BC cells, there are only some possibilities that NF-κB-EpCAM and c-Met-NF-κB may have cross talk, and, to become clear, further studies are needed.

## PI3K/Akt/mTOR signaling pathway and its importance in cancer

PI3K/Akt and mammalian target of rapamycin (mTOR; PAM) pathways are two signaling pathways that are necessary for many cellular activities, such as motility, proliferation, differentiation, metabolism, survival, and angiogenesis, in both normal physiology and development of cancer [75]. For as much as these pathways are closely connected to each other, they are often regarded as a unique pathway (PAM) [76, 77]. Phosphorylated tyrosines of receptor tyrosine kinases bind to PI3-kinase. This enzyme then phosphorylates the membrane lipid phosphatidylinositol-4,5-bisphosphate (PIP2) into phosphatidylinositol-3,4,5-trisphosphate (PIP3). Thus, PIP3 phosphorylates Akt, which is a serine/threonine kinase. Another crucial Akt activator is mTOR complex 2 (mTORC2). Activated Akt phosphorylates several substances, including mTORC1 and inhibitors (Tsc2 and PRAS40) to relieve the inhibition of mTORC1 kinase activity; thus, it stimulates protein synthesis, resulting in cell enlargement [78, 79]. Akt also inhibits apoptosis by deactivating pro-apoptotic protein Bcl-2 antagonist of cell death (BAD), as well as by promoting the breakdown of p53 as a pro-apoptotic protein via activation of E3 ubiquitin-protein ligase MDM2 [79].

PI3K/Akt/mTOR pathway has an integral role in cancer cells; for example, it has been estimated to be activated in 70% of all BCs [77, 80]. Akt phosphorylation, which demonstrates PAM activation, occurs in 50% to 70% of NSCLCs and 30% to 66% of head and neck squamous cell carcinomas (HNSCCs) [81, 82]. This pathway activation is also responsible for targeted therapy resistance in many cancer types [83].

### EpCAM and PI3K/Akt/mTOR

Downregulation of PI3K/Akt/mTOR signaling pathway proteins occurs subsequent to EpCAM silencing and is associated with the decreased colony formation, proliferation, and cellular invasion in prostatic cancer cells [84]. EpCAM-regulated carcinogenesis was proved to be associated with PI3K/Akt/mTOR signaling pathway activation in animal experimentation on prostatic cancer [84]. Consequently, this implies that EpCAM expression is closely associated with activation of this pathway in tumorigenesis. Another study showed that the roles of the Akt signaling pathway in EpCAM were related to tumorigenesis. It revealed that nasopharyngeal carcinoma cell lines overexpressing EpCAM had a significant increase in the level of crucial molecules of this pathway, such as mTOR and activated Akt. Also, after treatment of this cell line with the Akt inhibitor, MK2206 or the mTOR inhibitor (rapamycin) reduced the expression of Vimentin and SLUG (as EMT biomarkers). Thus, EpCAM induced invasiveness. Also, it confirms that activation of the Akt/mTOR signaling pathway is necessary for EMT, resulted from EpCAM overexpression in nasopharyngeal carcinoma cells [85].

### HGF/c-Met and PI3K/Akt/mTOR

PI3K/Akt/mTOR is also one of the pathways that controls c-Met regulated cell proliferation, survival, and migration [86]. Also, phosphorylated (activated) c-Met leads to phosphorylation of PI3K and RAS. Thus, it activates PI3K pathway indirectly later [79]. A new role for c-Met activated PI3K axes is involved in the constitution of lamellipodial protrusions that are crucial for transmigration cells through endothelial cells. This requires the mobilization of Rac1, p47phox, and localized reactive oxygen species (ROS) production [87]. It has also been shown that c-Met/PI3K/Akt signaling is responsible for resistance to photodynamic therapy and doxorubicin in carcinomas via enhancement of BCRP/ABCG2 expression [88]. Concurrent overexpression of c-Met and Akt leads to synergistic activity of these proto-oncogenes, resulting in rapid cancer expansion [89].

### HGF/c-Met, EpCAM cross talk in PI3K/Akt/mTOR

It is well established that the c-Met/PI3K/Akt signaling axis is crucial to regulate tumor cell critical functions, such as proliferation and survival. It is well established that c-Met uses the PAM axis to regulate tumor cells’ critical functions, such as proliferation and survival; on the other hand, some studies have proven that EpCAM uses the same axis for carcinogenesis. Although different outcomes are expected regarding which antigen has triggered the pathway, it could be suggested that these two TAAs interact through this signaling axis (Fig. 2).

## Wnt signaling pathway and its importance in cancer

Three different Wnt axes have been characterized, i.e., Ca, planar cell polarity (PCP), and canonical β-catenin/T cell factor 1 (TCF-1) pathways. The two latter branches are considered to antagonize each other. Once the canonical axis initiates, before nuclear translocation, β-catenin protein aggregates in the cytoplasm; then, at the level of the genome, it binds to the specific transcription factors to trigger the expression of specific genes [90]. Manifold localization of β-catenin in the nucleus and cytosol is identified in human BC frequently [91]. In the PCP axis, JUN-N-terminal kinase and GTPases are involved in regulating c-Jun-dependent transcription and cell migration. The Ca axis is associated with activation of phospholipase C or GMP-specific phosphodiesterase, followed by the release of intracellular Ca into the cytosol to activate downstream signaling proteins consisting of CREB and NFAT transcription factors mainly to regulate migration [90, 92]. One of the most critical signaling axes in embryonic development is the Wnt pathway, which regulates cell self-renewal, differentiation, proliferation, and migration [90]. This axis is generally silent until cell regeneration is needed in stem cells [93]. Hyperactive Wnt signaling can lead to aberrant cell proliferation and has been signified in the pathogenesis of BC, CRC, melanoma, and leukemia [94]. It is activated in more than 50% of BCs and is associated with decreased overall survival [95, 96]. Of note, the significance of the Wnt axis role in triggering, progression, or maintenance of distinct BC subtypes is controversial [96].

### EPCAM and Wnt pathway

Several different studies have indicated that EpCAM causes tumorigenesis using the Wnt axis. EpCAM signaling is activated when EpCAM is cleaved into EpEX and intracellular domain (EpICD) by specific enzymes. This cleavage only occurs when a temporary proliferation is required. EpEX sheds outside the cell, and EPICD is released to the cytoplasm. Four and one-half LIM domain protein 2 (FHL2) is a cytosolic protein, which is an interaction partner for EpICD and a co-activator of β-catenin [97, 98]. In a study by Yamashita et al. [99], EpCAM was found as a novel Wnt axis target gene, which could be considered as a biomarker for this pathway activation. They found a positive correlation between the EpCAM expression level and Wnt signaling genes, such as BAMBI and DKK. Also, it was discovered that activation of this pathway enriched the population of EpCAM+ cells [100]. In another study, it was detected that EpCAM overexpression in BC cell lines regulated Wnt axis components. The mRNA level of negative regulators of Wnt signaling (SFRP1 and TCF7L2) decreased in EpCAM-expressing cell lines after EpCAM overexpression; it means that Wnt signaling increased [101]. Transcription of EpCAM target genes, including c-Myc cyclins and TCF1, is activated as a result of translocation of β-catenin bound to FHL2 and EpICD into the nucleus [97, 98].

### HGF/c-Met and Wnt pathway

C-Met/HGF signaling has been shown to be an upstream regulator for the Wnt pathway [86, 102]. C-Met expression is controlled by the β-catenin signaling and downregulated by inhibition of Wnt signaling using Frzb and DNLRP5, two Wnt antagonists [103, 104]. C-Met and α3β1 integrin response to HGF and laminin, respectively, to regulate cell survival via the Wnt cascade [105]. Frizzled-8 (FZD8) as a Wnt receptor is known to be necessary for the interaction between the Wnt/β-catenin axis and c-Met since it is upregulated through the ERK/c-Fos cascade by c-Met in cancer stem-like cells [106]. Moreover, Wnt signaling is responsible for the development of acquired anti-c-Met therapy resistance since its components are upregulated in resistant cells [107].

### HGF/c-Met and EpCAM cross talking in the Wnt pathway

Several lines of evidence suggest that EpCAM and c-Met as signal transducers contribute to Wnt downstream effectors to initiate and develop cancer. It provides a logical explanation for the development of dual targeting drugs such as MM-131 a bispecific anti-MET/EpCAM mAb [108] and also further drug discoveries targeting concurrently all three c-Met, EpCAm and Wnt pathway simultaneously to overcome both innate and acquired resistance to tyrosin kinase inhibitors (TKIs).

## RAS/MAPK signaling pathway and its importance in cancer

Following the autophosphorylation of receptor tyrosine kinases, such as c-Met or many other receptor types, the activated receptor binds to the adaptor protein growth-factor-receptor-bound protein 2 (GRB2). GRB2 is then bound to the nucleotide exchange factor Son of sevenless (SOS); thus, phosphorylation of RTK recruits SOS to the plasma membrane, where RAS is also localized [109]. GTP-bound RAS (activated form) binds to RAF proteins (c-RAF, BRAF, and ARAF) and induces conformational alternations to dimerize RAF, which is activated and turns on the kinase cascade [110]. As mentioned above, RAS can also activate PI3K, triggering the PI3K/Akt/mTOR axis [111]. Activated RAF phosphorylates and activates mitogen-activated protein kinase kinases 1 and 2 (MEK1 and MEK2); these kinases are able to catalyze the phosphorylation of MAPKs, ERK1, and ERK2, thus rank-ordering the MAPK cascade from RAS, RAF, MEK, and finally to ERK [112, 113]. Eventually, the expression of immediate-early genes, c-fos, and c-Myc is upregulated [113]. Ultimately, ERK activates transcription factors involved in fundamental cellular processes, such as proliferation, apoptosis, differentiation, migration, adhesion, and cell polarity [114]. RAS/MAPK is one of the dominant cancer-initiating pathways and has a well-known impression in tumorigenesis by enhancing the survival and metastasis of cancer cells [115]. It is noteworthy that activating point mutations across any of the components of the pathway have been known as either trigger of cancer (mutations of RAF and RAS family genes) or as poor prognosis indicator (mutations of ERK and MEK) [114, 116]. Mutations in RAS genes, which make this enzyme continually active, are frequently detected in many cancer types; however, specific patterns exist between the cancer type and mutation frequencies associated with each RAS gene [113]. Mutations in RAF and RAS genes are considered the most common ones associated with chemotherapy and targeted therapy resistance [115].

### EPCAM and RAS/MAPK

It has been discovered that synergistic activation of this pathway by several factors (such as FGF, SCF, or HGF) stimulates the activation of the MAPK pathway [117]. In addition to RTKs and G-protein-coupled receptors, EpCAM has been considered as the RAS/MAPK cascade regulator. Gao et al. found that knockdown of EpCAM by small interfering RNA (siRNA) significantly reduced the expression and phosphorylation of RAS/MAPK components, p-ERK, p-RAF, and RAS in BC cells and repressed their malignant behavior [118]. In accordance with the latter report, phosphorylated ERK was decreased in EpCAM CRISPR/Cas9-mediated knockout cells, suggesting EpCAM augments integrin-mediated signaling [119]. Sankpal et al. defined a double-negative feedback loop between EpCAM and the activated MAPK pathway. They examined signaling pathway activity and EpCAM expression in a panel of 31 epithelial cancer cell lines; a strong inverse correlation between MAPK/ERK cascade activity and EpCAM expression was observed. To confirm their results, they used TGF-β1/TNF-α or EGF to induce MAPK/ERK activity; decreased EpCAM expression was subsequently observed [120].

### HGF/c-Met and the RAS/MAPK signaling pathway

As mentioned above, the RAS/MAPK pathway is also considered a downstream effector of HGF/c-Met signals, among other signal transduction pathways [86, 121]. Stimulated c-Met activates RAS to induce the expression of c-fos and c-Jun via the MAPK cascade in order to mediate cell scattering, invasion, and mobility [122]. The MAPK pathway is also responsible for HGF induced cell morphogenesis [123]. HGF induces the expression of ETS proto-oncogene 1, transcription factor (ETS1) by activation of ERK1. Activated ERK1 phosphorylates the threonine 38 residue of ETS1 to induce transcriptional activation [124]. Hypoxia-induced VEGF expression is known to be regulated by HGF/c-Met signaling via the MAPK axis [121]. Co-expression of Akt/c-Met stimulates the activation of the MAPK pathway as a result of the synergistic activity [125].

### C-Met and EpCAM cross talk in the RAS/MAPK pathway

**Fig. 3:**
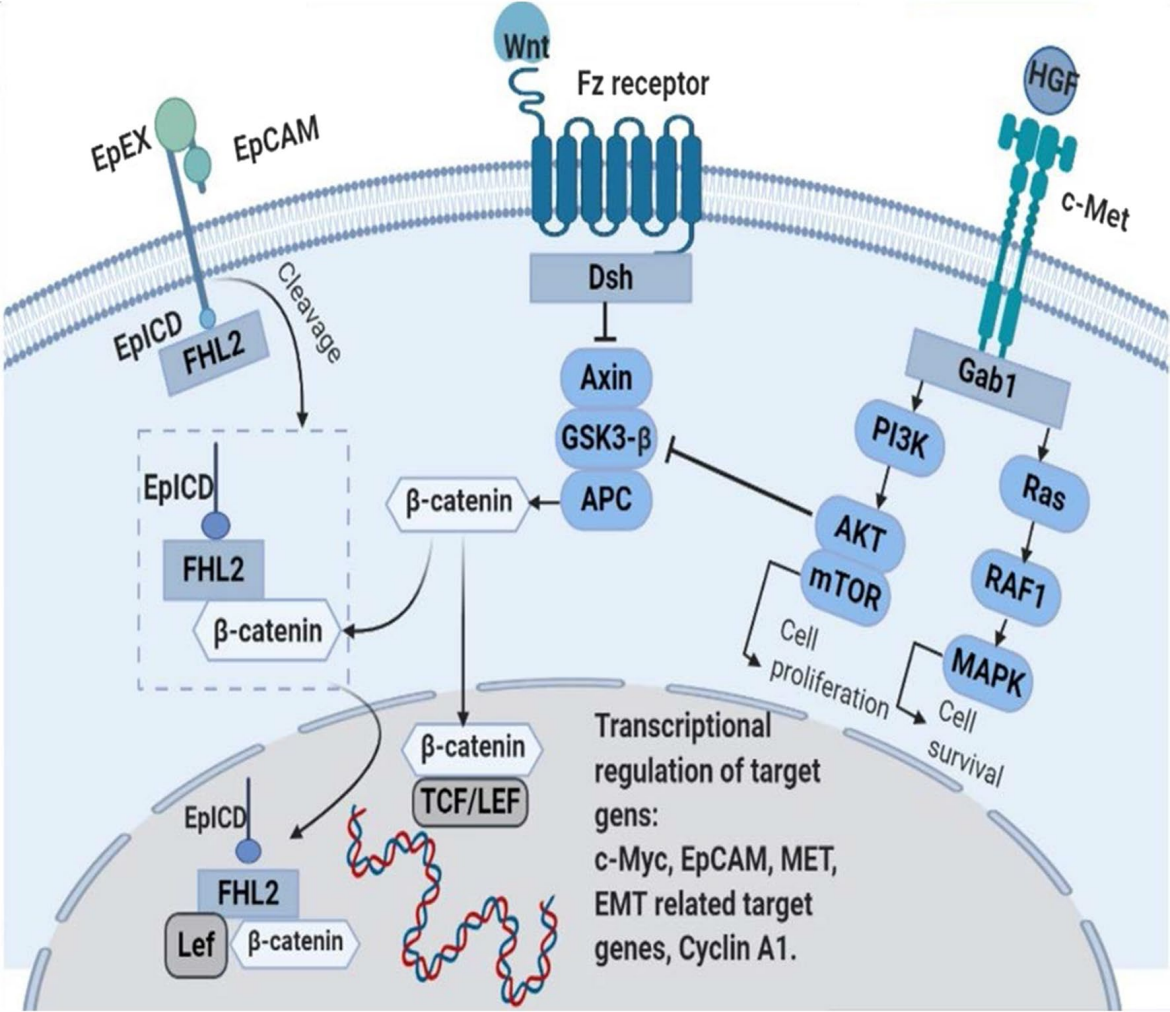
Cross talk between EpCAM, c-Met, and Wnt-β-catenin signaling pathways. Full-length EpCAM is cleaved, releasing EpCAM’s ectodomain (EpEX). Following the cleavage step, EpCAM’s cytoplasmic tail (EpICD) is released and associates with FHL-2 and β-catenin and translocates to the nucleus, upregulating transcription of EpCAM target genes via LEF consensus sites. C-Met activates MAP kinase (RAF/MEK/ERK) and PI3K/Akt signaling to induce cell proliferation and survival in cancer cells. C-Met and Wnt-β-catenin signaling pathways mainly cooperate in regulating EMT. C-Met contributes to nuclear translocation of β-catenin by its tyrosine phosphorylation or inhibition of the β-catenin degradation complex by Akt that phosphorylates glycogen synthase kinase-3β (GSK3β). This might, in turn, result in increased availability of non-bound β-catenin that may be stabilized by association with EpICD

The role of the MAPK cascade in the transduction of c-Met signaling into the nucleus is almost well defined; however, since research studies conducted on the cross talk between EpCAM and the RAS/RAF/MEK/ERK cascade have been limited to recent years and even sometimes are conflicting, it is almost impossible to define a conclusive opinion regarding the cross talk between c-Met and EpCAM through this pathway (Fig. 3).

## Resistance to targeted therapy in EpCAM and c-Met

### Drug resistance

Failure in cancer therapy can happen via two general causes. The first one is intrinsic in cancer cells; for example, cell membrane transporter proteins are important in drug resistance because they alter drug transport. ABC transporters (ATP-binding cassette) specially P-gp (P-glycoprotein) play an important role in clinical drug resistance by many of mechanisms for example high level expression by gene rearrangement. The other is the gradual genetic and epigenetic abnormalities that occur in cancer cells [126]. Tumors such as renal cancer, hepatocellular carcinoma, and malignant melanoma display intrinsic resistance [127]. In BC, it was seen that cancer therapy resulted in a stem cell-like phenotype in non-stem tumor cells [128]. In some cases, it was demonstrated that chemotherapy could potentially raise the levels of circulating endothelial progenitor cells (EPCs) that cause tumor growth and metastasis [129]. Researches have shown a positive correlation between chemotherapeutic resistance and the number of spontaneous genetic mutations [130]. Mutation in several genes confers resistance to cancer therapy. For example, in ovarian cancer, a mutation in the p53 gene was seen in platinum chemotherapy [131] and, in BC treated with anticancer drugs, reduced response to chemotherapy; most cases had a mutation in p53 [132]. Also, in head and neck cancer, polymorphism in p53 influenced clinical response to cisplatin-based chemoradiotherapy [133].

In metastatic colorectal cancer, mutations of K-Ras have an adverse effect on response to anti-EGFR antibody and cetuximab [134]; also, in NSCLC, mutations in K-Ras affect resistance to treatment with gefitinib or erlotinib drugs [135]. Gene amplification is the other way that confers resistance to cancer therapy. For example, an increasing copy number of the dihydrofolate reductase (DHFR) gene was seen in a patient that received methotrexate [136]. In breast cancer, a correlation between the copy number of the HER2 gene and sensitivity to Herceptin was seen [137]. Moreover, gene amplification is a way that in chronic myelogenous leukemia (CML) lead to over expression of the Bcr-Abl protein as a mechanism of acquired imatinib resistance [137]. The use of cetuximab in patients with colon cancer results in EGFR gene amplification and treatment resistance [134]. Another mechanism, which is a problem in treatment, is gene rearrangement; for example, in leukemia, rearrangements of the MDR-1 gene cause drug refractory [138]. Epigenetic changes are another obstacle in cancer therapy; for example, in patients with gliomas, methylation of the promoter of the DNA-repair enzyme O6-methylguanine-DNA methyltransferase (MGMT) is a problem in cancer therapy with alkylating agents [139] (Table 2).

**Table 2:** Genetic and epigenetic causes of drug resistance

Mutation in gene		
Cancer	Gene that causes resistance	Clinical data
Ovarian cancer	p53	Platinum chemotherapy
Breast cancer	p53	Chemotherapy
Head and neck cancer	p53	Cisplatin based chemoradiotherapy
Metastatic colorectal cancer	K-ras	Cetuximab
Non-small cell lung cancer (NSCLC)	K-ras	Gefitinib or erlotinib
Gene amplification		
Cancer	Gene that causes resistance	Clinical data
Breast cancer	HER2	Herceptin
Chronic myelogenous leukemia (CML)	Bcr-Abl	Imatinib
Colon cancer	EGFR	Cetuximab
	Dihydrofolate reductase (DHFR)	Methotrexate
Gene rearrangement		
Cancer	Gene that causes resistance	Clinical data
Leukemia	MDR-1	drug refractory
Epigenetic changes		
Gliomas	O6-methylguanine-DNA methyltransferase (MGMT)	Alkylating agents

#### Roles of EpCAM in drug resistance

Most studies have analyzed the relationship between high EpCAM expression in cancer cells and permanent proliferation signals, as well as overexpression of various targeted genes, including c-Myc and cyclins [140]. EpCAM has been identified as a cancer stem cell marker in solid tumors and has a significant correlation with all the characteristics of cancer stem cells, EMT, and metastasis [123]. Besides, EpCAM overexpression is related to Wnt/β-catenin pathway activation in cancers, which is a potential causal pathway for cytoplasmic and nuclear accumulation of β-catenin, indicating proliferation and reduced apoptosis in cancer cells [141]. EpCAM is applied as a suitable target in immunotherapy approaches, such as antibody-based approaches for in vitro/in vivo and ongoing clinical trials. Recent results have indicated that humanized anti-EpCAM antibodies are successful in both preclinical and early clinical studies and selectively targeted EpCAM-positive cell lines with a greater potency without any relapse [142]. However, despite these favorable achievements, anti-EpCAM monoclonal antibodies have not shown as much promise as initially indicated, and resistance to mAb has become a major obstacle recently. Several studies have shown that anti-EpCAM antibodies limit therapeutic efficacies as the results of showing dose- and target-dependent in metastatic cancers, short serum half-life, altered EpCAM expression pattern, and differential cleavage and localization of EpICD in cancer cells (Fig. 3) [143, 144]. Thus, larger clinical trials are necessary to approve novel antibodies in the treatment of specific tumors.

#### Role of c-Met in drug resistance

A variety of preclinical data have demonstrated that amplification of c-Met signaling occurs in many malignancies as a result of MET gene mutation or overexpression. Resistance to targeted therapeutics developed by the activation of c-Met kinase has appeared as a considerable mechanism of resistance in multiple types of cancers [145]. As depicted in Fig. 2, in c-Met signaling, overexpression of the c-Met receptor and HGF results in sustained PI3K/Akt/mTOR and MAPK signaling activation through Gab1 and mutation of *MET* kinase domain, which are associated with therapy resistance [87]. Additionally, c-Met signaling can also mediate aberrant localization and phosphorylation of β-catenin in cancer cells. Phosphorylated β-catenin translocates to the nucleus, binds to TCF/LEF transcription factors, and promotes the expression of many target genes, including those involved in cell proliferation, anti-apoptosis, invasion, and angiogenesis (Fig. 3) [146]. Overexpression of HGF and/or c-Met can contribute to therapy resistance in radiotherapy, chemotherapy, and targeted therapies (including EGFR inhibitors in NSCLC and CRC) [109, 147]. Since patients who developed EGFR mutant lung adenocarcinomas with acquired resistance to EGFR inhibitor drugs (gefitinib or erlotinib) exhibit increased copy numbers of MET, c-Met-HGF inhibitors are used for lung cancer treatment alone or in combination with other drugs [148]. Targeting c-Met has been investigated in anti-EGFR resistant cells because c-Met is a key player in anti-EGFR resistance [148]. The results showed that c-Met inhibitors could overcome anti-EGFR resistance and suppress resistant cell proliferation [148]. The important role of c-Met in anti-EGFR resistance promoted researchers to investigate the effects of EGFR and c-Met inhibitors [149]. The findings showed that using bispecific Ab (anti-EGFR and anti-c-Met) could suppress growth in anti-EGFR resistant cells more efficiently compared to anti-EGFR agents, such as gefitinib, erlotinib, and so on [149]. As can be seen, the results of different studies demonstrate the efficacy of c-Met targeting; in the following, we will discuss the advantages of targeting c-Met and EpCAM.

#### Targeting c-Met and EpCAM: a way to overcome drug resistance

It has been well established that aberrantly expressed EpCAM has a direct impact on the cell cycle, proliferation, and upregulation of the oncogenic transcription factors c-Myc and cyclin A/E [150]. As a matter of fact, the intracellular part of EpCAM (EpICD) is crucial and sufficient to upregulate proto-oncogenes activation [151]. Moreover, c-Myc alterations contribute to acquired resistance to c-Met inhibitors in different MET-overexpressed cancers [152]. It is conceivable that targeting c-Met, along with other aberrant proto-oncogenes, can modulate c-Myc expression and cell survival in EpCAM-positive and overexpressed c-Met-resistant cancer cells [102, 153]. In addition, several studies have shown co-overexpression of EpCAM and c-Met pathways and their significant interaction in developing resistance to currently targeted therapies in cancer tissues. Similar to EpCAM, c-Met also has a putative role in the acquisition of the stem cell status and EMT in cancer that causes tumor cell dissociation and metastasis [154]. Understanding different resistance mechanisms to currently-existing inhibitors can provide new insights into combinatorial targeting of the driver oncoprotein and further downstream effectors to avoid or postpone the therapy resistance. For instance, MM-131, a bispecific anti-MET/EpCAM mAb, has been developed to inhibit cell proliferation and migration in c-Met positive/HGF-positive tumors, which also overexpresses EpCAM [109]. Thus, concurrent administrations of novel agents against c-Met and EpCAM are recommended to implement optimal efficacy in current treatments.

Undoubtedly, reviewing existing evidence and mechanisms regarding therapy resistance confirms the structural and functional intricacy of aberrantly expressed EpCAM and c-Met, which makes them function as proto-oncogenes in several malignancies. Nevertheless, it is still needed to be elucidated whether these two oncogenic signaling pathways possess any initiating roles in the development of cancer or they are merely contributing molecules in the propagation of tumors or development of resistance to existing therapeutic agents. As we discussed in this review, the upregulation of EpCAM and c-Met takes place in many human carcinomas and is in close relation to the development and propagation of cancers; thus, suppressing overexpression of EpCAM and c-Met may represent a potential potent therapeutic approach. In addition, their cross talks with other oncogenic pathways result in amplification of their underlying signaling cascades and resistance development to different targeted therapeutic agents.

## Novel therapy targeting for c-Met and EPCAM

### C-Met and novel therapy

Anti-c-Met agents can be classified into four types: selective c-Met tyrosine kinase inhibitors (TKIs), non-selective c-Met inhibitors, monoclonal antibodies against HGF/c-Met, and microRNAs (miRNAs). Selective c-Met inhibitors have a very high selectivity (more than ten thousand times) for c-Met compared to other kinases [155]. However, this selectivity does not mean that other tyrosine kinases are not inhibited at all. Volitinib (Savolitinib), SAR125844, tepotinib (EMD1214063), capmatinib (INCB28060), AMG337, Indo5, tivantinib, and PHA665752 are of such selective c-Met inhibitors [156–160], which showed anti-tumor effects in various cancers with high MET expression, including gastric and hepatocellular carcinoma, papillary renal carcinoma, and NSCLC [158, 161]. Volitinib, SAR125844, and PHA665752 have anti-proliferative and pro-apoptotic effects by inhibition of RAS/MAPK and PI3K/Akt signaling pathways; meanwhile, tepotinib inhibits c-Met signaling in both HGF-dependent and independent ways [156].

One way to improve the response to TKIs in patients with lower c-Met overexpression is to expand the target kinases [162]. Non-selective c-Met inhibitors include crizotinib (PF-02341066), foretinib (XL-880), cabozantinib (XL-184), glesatinib (MGCD265), BMS-777607, and MK2461, which in addition to c-Met, can inhibit other tyrosine kinases, such as ALK, RON, VEGFR2, KIT, TIE2, PDGFR, VEGFR1, VEGFR3, RET, and FLT-3 [163–165], through binding to the ATP binding site on tyrosine kinases. The considerable effects of non-selective TKIs, especially crizotinib, led to the US Food and Drug Administration (FDA) approval of crizotinib in ALK+ cancers [166]. VEGFR inhibitors are also capable of inhibiting angiogenesis along with TKIs benefits. The combination of non-selective TKIs with chemotherapy and checkpoint inhibitors has been investigated in various trials with promising results [162]. The point that should be considered about non-selective TKIs is that the anti-tumor function of these inhibitors might be higher than that of selective c-Met inhibitors due to inhibition of multiple kinases. Meanwhile, the toxicity of non-selective TKIs is higher than selective c-Met inhibitors, limiting their prescribed dosage [167]. In some cases, high toxicity and damage of non-selective TKIs to various organs might be comparable to those caused by the tumor itself. Therefore, it is better to use specific TKIs based on the overexpressed tyrosine kinase in each cancer type [165].

Various monoclonal antibodies have been developed to block c-Met/HGF signaling pathways, some of which competitively bind to c-Met, and the others are served as traps for c-Met, preventing ligand attachment and dimerization of c-Met [168]. Onartuzumab (MetMAb), SAIT301, ABT-700 (h224G11), ARGX-111, and DN30 are specific antibodies against c-Met, preventing the binding of c-Met to HGF [169–171]. SAIT301 also internalizes and destroys c-Met after binding to it [171]. The mechanism of ARGX-111 is through stimulating antibody-dependent cellular cytotoxicity (ADCC) in cells with excessive c-Met expression [172]. DN30 is involved in decreasing the c-Met signaling pathway through a variety of ways, including c-Met destruction, reducing c-Met expression, c-Met proteolytic detachment, and shedding c-Met from the cell surface, providing traps for HGF [162, 170]. Thus, DN30 with dual effects on c-Met and HGF separately can inhibit the c-Met/HGF pathway [173].

Bispecific antibodies with different specifications are an interesting option in targeted therapy. Emibetuzumab (LY2875358) against c-Met and HGF and MP0250 against HGF and VEGF are two bispecific antibodies that prevent c-Met attachment to the ligand, increase the internalization, destroy c-Met molecules, and thereby reduce the signaling of the c-Met pathway [149, 174, 175]. Moreover, LY3164530 and JNJ-61186372 bispecific antibodies bind to c-Met and EGFR and have shown good results in inhibiting tumor growth [176, 177].

HGF targeting antibodies, including rilotumumab (AMG-102), ficlatuzumab, TAK-701, and YYB-101, bind to HGF and prevent c-Met attachment, leading to the induction of synergistic anti-tumor responses combined with other TKIs and EGFR inhibitors [162, 172, 178, 179]. Clinical trials have examined the safety and efficacy of these antibodies in cancers and have shown that combinational therapy with other treatments has improved patients’ responses and their survival [169, 178, 179].

Nowadays, one of the new methods of targeted therapies is based on miRNAs. The expression of miRNAs in cancerous tissue is different from normal tissue, leading to cancer progression, tumor growth, invasion, and metastasis [180]. Some of these miRNAs target c-Met by increasing or decreasing its signaling and play a role in tumor progression or suppression. For example, miR-93 activates c-Met and PI3K/Akt signaling pathways and leads to tumor progression and invasion [181]. However, miR-101, miR-206, and miR-26a target c-Met/HGF and inhibit tumor growth and metastasis [182–184]. Therefore, the use of either miRNA antagonists, agonists, or miRNA mimics that inhibit c-Met signaling is a potential and promising treatment for cancer [165].

Taken together, c-Met could be targeted using selective and non-selective inhibitors, monoclonal or bispecific antibodies, and miRNAs to inhibit tumor progression. Selective inhibitors have more specificity and low adverse events compared to non-selective ones. The clinical trials on c-Met inhibitors are still at the beginning of the way and require further evaluation.

#### EpCAM and novel therapy

EpCAM (CD326) is found only in the basolateral membrane of epithelial tissue while overexpressed throughout the cancerous tissue membrane, making this molecule a good candidate for targeting in the treatment of cancer [185]. Tight junctions hamper the targeting of EpCAM in normal tissues and prevent unspecific tissue damage [186]. Due to the loose adhesion of EpCAM compared to other CAMs, increased expression of EpCAM is associated with invasion, metastasis, and poor prognosis in many cancers, including basal-like and luminal B BCs [185, 187, 188].

The first anti-EpCAM monoclonal antibody was a mouse antibody developed in 1979 called edrecolomab [189]. Edrecolomab causes cytotoxicity for the tumor through cell-dependent mechanisms, such as ADCC and complement-dependent cytotoxicity (CDC) [190, 191]. Despite its benefits in early clinical trials, the trials were halted due to limited efficiency and several side effects of the murine and chimeric types [142, 192, 193]. Studies have suggested that the inefficiency of edrecolomab is due to its low affinity to EpCAM. Two high-affinity antibodies, 3622W94 and ING-1, were developed but bound to all EpCAM-expressing cells, leading to side effects such as acute pancreatitis in clinical trials [142]. Adecatumumab, which has a moderate affinity to EpCAM, can target tumor cells with high EPCAM expression and reduce aggression and metastasis in patients with fewer side effects [194]. In addition to ADCC and CDC, one of the therapeutic mechanisms of adecatumumab is through inhibition of tumor cell metabolism [142]. There are specific conditions needed for an efficient ADCC or CDC, including enough number of antibodies attached to the target cell, a sufficient number of effector immune cells (such as natural killer [NK] cells, monocytes, and granulocytes), and the absence of ADCC suppressor cells (such as regulatory T cells [Tregs] and myeloid-derived suppressor cells [MDSCs]) in the tumor microenvironment. Moreover, the internalization of EpCAM and bounded antibodies pose challenges to ADCC/CDC. Therefore, it seems that other mechanisms should be considered in designing anti-EpCAM antibodies [143].

The next generation of EpCAM-targeting antibodies is based on closing immune cells to tumor cells. Catumaxomab is a bispecific antibody against EpCAM and CD3, which brings T cells closer to EpCAM+ cells. Besides, the fragment crystallizable (Fc) of the antibody can bind to Fc receptors (FcRs) on the surface of immune cells and induce ADCC, making it a three-functional antibody (Triomab) [195, 196]. Fc also binds to FcRs on the surface of DCs and connects them to T cells to activate T cells [197]. In clinical trials, catumaxomab showed promising efficacy by increasing anti-tumor cytokines and had low toxicity that led to its approval [195, 196].

Another bispecific molecule is the bispecific T cell engagers (BiTEs), consisting of two single-chain variable fragments with different specificities [198]. Solitomab or MT-110 is a BiTE against EPCAM and CD3, which bridges between tumor cells and T cells, leading to lysis of tumor cells [196, 198]. The advantages of this molecule are smaller size and greater penetration into solid tumors compared to conventional antibodies [143]. Another way to activate immune cells, including T and NK cells, is to use IL-2 linked to an anti-EpCAM antibody. Tucotuzumab celmoleukin (EMD 273066 or huKS-IL2) is an immunocytokine consisting of tucotuzumab (anti-EpCAM antibody) and IL-2 (celmoleukin) [199]. The combination of huKS-IL2 with cyclophosphamide prevents Treg proliferation and increases anti-tumor effects [144]. An important point to consider when using T cell-involving antibodies is that the tumor microenvironment is immunosuppressive, and the infiltrated T cells are mostly exhausted. Therefore, simultaneous targeting of immunosuppressive agents, such as IDO and immune checkpoints (including PD-1 and CTLA-4), improves anti-tumor response [186, 196, 198, 200].

A common way to kill EpCAM+ tumor cells is to use immunotoxins. This means that EpCAM-specific antibodies are conjugated with cytotoxic agents, such as doxorubicin, paclitaxel, calicheamicin, topoisomerase I inhibitors, alpha amanitin, and indolinobenzodiazepine pseudodimers (IGNs), to specifically kill the EpCAM+ tumor cells. Antibodies enter the cell to release the toxin in the cytoplasm; thus, the extent of antigen expression, antibody affinity, and the ratio of toxic agents conjugated with each antibody is effective in its performance [201, 202]. Some of these substances have direct cytotoxic effects, and some others, such as fungal derivative alpha amanitin, induce apoptosis in tumor cells [203]. Oportuzumab monatox (VB4-845) and citatuzumab bogatox (VB6-845) are fusion proteins comprised of an anti-EPCAM single-chain variable fragment (ScFv) or Fab bound to pseudomonas endotoxin A or non-immunogenic toxin of Bougainvillea spectabilis. These fusion proteins enter EpCAM+ cells, and the conjugated toxin inhibits protein synthesis leading to cell apoptosis. Studies have reported tolerability and acceptable side effects of toxin-conjugated fusion proteins, as well as great anti-tumor effects in bladder, head and neck cancers, and epithelial tumors, which has led them to the next stages of clinical trials [204–206].

One of the problems with using antibodies is their large size; thus, researchers are trying to increase the tumor permeability by replacing antibodies with smaller peptides and aptamers [143, 207]. Using specific peptides instead of antibodies could solve this limitation (large size) and enhance the tumor permeability. Two protein families are mostly used in targeted therapy: (1) designed ankyrin repeat proteins (DARPins), a type of non-immunoglobulin-engineered protein derived from the ankyrin protein and (2) macrocyclic peptides produced by the random non-standard peptides integrated discovery (RaPID) system. These two protein families have high specificity and affinity to their targets, as well as high resistance to proteases. The EpCAM-specific types of these peptides have shown promising anti-tumor results and are being further investigated [208, 209].

Aptamers, also known as chemical antibodies, are single-stranded oligonucleotides that shape a three-dimensional structure and bind to their targets just like antibodies [210]. Aptamers have different advantages over antibodies, including much smaller size (making them more appropriate to penetrate solid tumors), less immunogenicity (reducing resistance to treatment), the capability to manipulate their length and physicochemical properties (making them possible to change the half-life, pH sensitivity, flexibility, and conjugation capacity), fast and simple production procedure, negligible inter-batch variation, and better temperature resistance [210–214]. Xiang et al. showed that EpCAM-specific aptamers had better biodistribution and had up to four times high tumor penetration, and therefore better performance than anti-EpCAM antibodies [215]. Similar to antibodies, one of the applications of EpCAM-specific aptamers is to deliver cytotoxic molecules, such as doxorubicin and neocarzinostatin, to target cells [216]. The interesting point about the binding of drugs to aptamers is that by changing the length of the aptamer and its physicochemical features, the number of conjugating molecules can be increased without any particular effect on the affinity and specificity of the aptamer [211, 216]. The application of aptamers also faces challenges that are mainly related to their small size and nucleotide nature. These challenges include degradation by cellular nucleases, high renal clearance, and low penetration in biological membranes due to their negative charge. These challenges can be met by nucleotide modifications and choosing conjugates with appropriate molecular weight and positive charge [213, 214].

Moreover, EpCAM is associated with some miRNAs and genes involved in tumor progression [217]. The high levels of miR-130, miR-181, miR-17-92, and miR-92b have been associated with EpCAM overexpression in hepatocellular carcinoma [218, 219]. Further, miR-181 and miR-130 are overexpressed in EpCAM+ cells than in EpCAM cells and cause stemness features in EpCAM+ tumor cells. Hence, these miRNA families are associated with cancer stem cells in EpCAM+ tumors, and their inhibition reduces tumor cell proliferation.

To sum up, there are several generations of anti-EpCAM antibodies with different effector mechanisms, including ADCC, CDC, T-cell engaging, providing activatory cytokines for immune cells, and delivering toxins to the tumor cells. Although low-affinity mAbs have low efficacy, high-affinity mAbs might demonstrate more adverse events due to binding to all EpCAM+ cells. Researchers are trying to overcome the low-penetration issues by using ScFvs, BiTEs, aptamers, DARPins, RaPID system, and miRNAs instead of full-sized antibodies. The clinical trials on combining EpCAM targeting with other immunotherapies (such as checkpoint blockade) are ongoing.

## Conclusion and prospective

As mentioned before, tumors, stroma, and the immune system communicate with each other and contribute to tumor resistance. Furthermore, tumor cells are capable of escape from the immune system. TAAs on tumor cells deceive the immune system, leading to inappropriate and inadequate responses. EpCAM and c-Met are not only considered two prominent tumor-associated antigens but also expressed on healthy tissues. However, their overexpression on tumor cells results in over-activation of certain intercellular signaling pathways of proliferation, migration, and invasion of tumor cells. Subsequent the HGF coupling with c-Met as a tyrosine kinas receptor, angiogenesis could be initiated through migration and invasion to adjusting tissues. EpCAM is also associated with NF-κB signaling pathway activation leading to IL-8 production, which is one of the key mediators in angiogenesis. Hence, targeting both of them simultaneously might be a promising approach to hinder therapeutic resistance by blocking all potential alternative routes. Indeed, the investigation of cross talk between mentioned molecules and tumor stroma may shed light on finding new targets and could provide beneficial information for the rational design of a novel therapeutic approach in cancer.

## Data Availability

Not applicable.

## Abbreviations

NF-κB: Nuclear factor kappa-light-chain-enhancer of activated B cells
STAT3: Signal transducer and activator of transcription 3
c-MET: Mesenchymal epithelial transition factor (MET) or hepatocyte growth factor receptor (HGFR)
EpCAM: Epithelial cell adhesion molecule
COX2: Cyclooxygenase-2
MMP9: Matrix metallopeptidase 9
IKK: IκB kinase
PI3K: Phosphoinositide 3-kinase
AKT: Protein kinase B or (PKB)
ERK: Extracellular signal-regulated kinase
MAPK: Mitogen-activated protein kinase
EGFR: Epidermal growth factor receptor tyrosine kinase
EMT: Epithelial–mesenchymal transition
BCL2: B-cell lymphoma 2
BCLxL: B-cell lymphoma-extra large
XIAP: X-linked inhibitor of apoptosis protein
ER: Estrogen receptor
PR: Progesterone receptor
IGFIR: Insulin-like growth factor
VEGFR: Vascular endothelial growth factor receptor
c-Myc: Myelocytomatosis
IKB: Inhibitor of kB
